# Comparative Meta-Analysis of Carbohydrate Antigen 125 (CA125), Human Epididymis Protein 4 (HE4), and Diagnostic Indices (Risk of Malignancy Index (RMI) and Risk of Ovarian Malignancy Algorithm (ROMA)) for Pre-operative Detection of Ovarian Carcinoma

**DOI:** 10.7759/cureus.82415

**Published:** 2025-04-17

**Authors:** Adarsh Kumar Singh, Pooja Yadav, Manu Shukla, Shivani Samaiya, Sonali Singh, Shreya Sorout

**Affiliations:** 1 Department of Neurological Surgery, Centre of BioMedical Research (CBMR), Sanjay Gandhi Postgraduate Institute of Medical Sciences (SGPGIMS), Lucknow, IND; 2 Department of Obstetrics and Gynaecology, Maharani Laxmi Bai (MLB) Medical College, Jhansi, IND

**Keywords:** ca-125, he 4, ovarian carcinoma, risk of malignancy index (rmi), risk of ovarian malignancy algorithm (roma)

## Abstract

Ovarian cancer remains a leading cause of gynecological cancer-related mortality, primarily due to late-stage diagnosis. Effective pre-operative differentiation between benign and malignant ovarian masses is crucial for improving patient outcomes. This meta-analysis evaluates the diagnostic performance of carbohydrate antigen 125 (CA125), human epididymis protein 4 (HE4), the Risk of Malignancy Index (RMI), and the Risk of Ovarian Malignancy Algorithm (ROMA) by assessing their sensitivity, specificity, and overall accuracy. A systematic review of studies published between 2000 and 2024 identified 12 eligible studies with sample sizes ranging from 84 to 456 participants. Statistical analyses, including bivariate modeling and summary receiver operating characteristic curves, were performed. CA125 demonstrated the highest sensitivity (0.82) but had a high false-positive rate (0.357), limiting its specificity. HE4 exhibited higher specificity (0.171) and an improved diagnostic odds ratio (DOR = 17.00) compared to CA125. The ROMA and RMI showed comparable performance, with the ROMA achieving the highest area under the curve (AUC = 0.8619), followed by HE4 (AUC = 0.8586) and RMI (AUC = 0.8508), while CA125 had the lowest AUC (0.8128), indicating lower standalone diagnostic reliability. The findings suggest that HE4, ROMA, and RMI outperform CA125 in terms of specificity and overall diagnostic accuracy, with HE4 and ROMA particularly demonstrating superior specificity. These results support a multimodal diagnostic approach that integrates multiple biomarkers and risk indices to enhance pre-operative ovarian cancer detection and optimize patient management.

## Introduction and background

Introduction

Ovarian cancer has been estimated to be the third most common cancer among Indian women. According to the Global Cancer Statistics 2022 (GLOBOCAN 2022) Fact sheet, ovarian cancer is the 18th most common cancer worldwide [1]. The risk factors include older age, genetic predisposition, late menopause, nulliparity, hormone replacement therapy, tobacco smoking, high dietary fat, obesity, nuclear radiation exposure, and infertility. On the basis of epithelial appearance, ovarian carcinoma has been divided into five major categories: high-grade serous carcinoma (most common, around 68%), clear cell carcinoma (12%), endometrioid carcinoma (11%), mucinous carcinoma (3%), and low-grade serous carcinoma (3%) [2]. The five-year survival rate of ovarian cancer patients is 94% when diagnosed in Stage I, but unfortunately, only 15% of cases are diagnosed at this stage. A vast majority (62%) of cases are diagnosed only when cancer reaches Stages III and IV. The five-year survival at this point is only 28% [3].

The survival rate also varies according to the different histological types of the carcinoma [4]. The diagnosis of ovarian cancer is often delayed due to the vague presenting symptoms like bloating, pelvic or lower abdominal pain, early satiety, constipation, urinary symptoms such as urgency or frequency, and sometimes a palpable lump in the abdomen. Complete tumor resection during the primary cytoreductive surgery is one of the most important prognostic factors in ovarian carcinoma. Research articles suggest that ovarian cancer patients who are operated by gynecologic oncology surgeons tend to have a better survival rate than those who are operated by general gynecologists [5,6]. This fact itself highlights the importance of pre-operative differentiation of malignant from benign cases to improve the final outcome of surgery when done in a planned manner in the presence of a gynae-onco surgeon. The currently widely used modalities for the diagnosis of ovarian cancer include markers like carbohydrate antigen 125 (CA125), human epididymis protein 4 (HE4), and algorithms like Risk of Ovarian Malignancy Algorithm (ROMA) and Risk of Malignancy Index (RMI).

CA125

CA125 is an antigenic tumor marker expressed by epithelial ovarian neoplasms and the cells lining the endometrium, fallopian tubes, pleura, peritoneum, and pericardium [7,8]. A drawback of using CA125 as a tumor marker is that it also becomes elevated under certain physiological conditions like menstruation [9] and other benign conditions like fibroids and pelvic endometriosis. The levels of CA125 are raised in about 80% of women with epithelial ovarian cancer, but only in 50% of those with early-stage disease [10]. However, about 20% of patients with ovarian cancer do not express CA125 [11].

HE4

HE4 is encoded by gene WFDC2 and is overexpressed in ovarian cancer [12]. HE4 has showed sensitivity and specificity values higher than CA125 [13].

ROMA

The ROMA has been proposed to differentiate benign pelvic masses from epithelial ovarian cancer. It combines the levels of CA125 and human epididymal protein 4 (HE4) with menopausal status. Several studies have proved that with high-sensitivity and high-specificity ROMA can be a better marker to predict a malignant ovarian mass [14,15].

RMI

In 1990, Jacobs et al. initially proposed the RMI as ultrasound findings × serum CA125 level × menopausal status (1 for pre-menopausal and 3 for post-menopausal women). The RMI has been found to be the most effective and often used in clinical practice [16].

This meta-analysis was conducted to address the following research question: "Among the diagnostic tools CA125, HE-4, ROMA and RMI, which demonstrates the highest accuracy in distinguishing benign from malignant ovarian tumors, and how do these performances vary across menopausal subgroups?"

Hypothesis

We hypothesize that HE-4, ROMA, and RMI outperform CA125 in overall diagnostic accuracy, particularly in terms of specificity and diagnostic odds ratio (DOR), and that menopausal status influences marker performance.

Objective

The objective of this review is to evaluate and compare the diagnostic performance of CA125, HE4, ROMA, and RMI in the pre-operative diagnosis of ovarian carcinoma, by analyzing their sensitivity, specificity, and overall accuracy through a comprehensive meta-analysis of existing studies.

Materials and methods

Search Strategy

A comprehensive literature search was conducted using PubMed, MEDLINE, and Google Scholar to identify studies published between January 1, 2000 and February 29, 2024. The search focused on diagnostic performance evaluation of CA125, HE-4, ROMA and RMI in ovarian cancer. Due to institutional access limitations, Embase, Cochrane Library, and Scopus could not be searched, which is acknowledged as a limitation.

Search strings used have been described as follows:

PubMed/MEDLINE

("ovarian cancer" OR "ovarian neoplasm") AND

("CA-125" OR "cancer antigen 125" OR "HE4" OR "human epididymis protein 4" OR "ROMA" OR "risk of ovarian malignancy algorithm" OR "RMI" OR "risk of malignancy index") AND

("diagnosis" OR "diagnostic accuracy" OR "sensitivity" OR "specificity")

Google Scholar

"ovarian cancer diagnosis" AND ("CA-125" OR "HE4" OR "ROMA" OR "RMI") AND ("sensitivity" OR "specificity" OR "meta-analysis")

Filters applied include human studies, English language, and women only. Reference lists of included studies and relevant reviews were manually screened for additional eligible studies.

Study Selection

The study selection was done following the Preferred Reporting Items for Systematic reviews and Meta-Analysis (PRISMA) 2020 flow diagram and included four main phases described as follows:

Identification*:* Two thousand two hundred and forty-six records were identified through database search.

Screening*:* One thousand six hundred and eighty-eight records were screened after removal of duplicates and automation tools.

Eligibility*:* Two hundred and fifty-one studies remained after abstract screening.

Inclusion*: *Twelve studies were included after full-text evaluation.

A total of 177 full-text articles could not be retrieved, primarily due to subscription restrictions and lack of author response, which is acknowledged as a limitation.

The exclusion and inclusion criteria for the studies are listed in Table 1.

**Table 1:** Inclusion and exclusion criteria for studies included in the study CA125: Carbohydrate antigen 125; HE4: Human epididymis protein 4; ROMA: Risk of Ovarian Malignancy Algorithm; RMI: Risk of Malignancy Index

Serial Number	Inclusion Criteria	Exclusion Criteria
1.	Studies evaluating the diagnostic performance of CA125, HE4, RMI, or ROMA in ovarian carcinoma for pre-operative diagnosis.	Studies that do not provide original data (e.g., reviews, editorials).
2.	Studies that provide data on sensitivity, specificity, positive predictive value (PPV), and negative predictive value (NPV).	Studies with a sample size too small (< 40 patients) to provide reliable estimates.
3.	Peer-reviewed articles, clinical trials, or observational studies with a clear focus on ovarian carcinoma.	Studies including only a specific population based on menopausal status.
4.	-	Non-English language articles or those without full-text availability.

**Figure 1:**
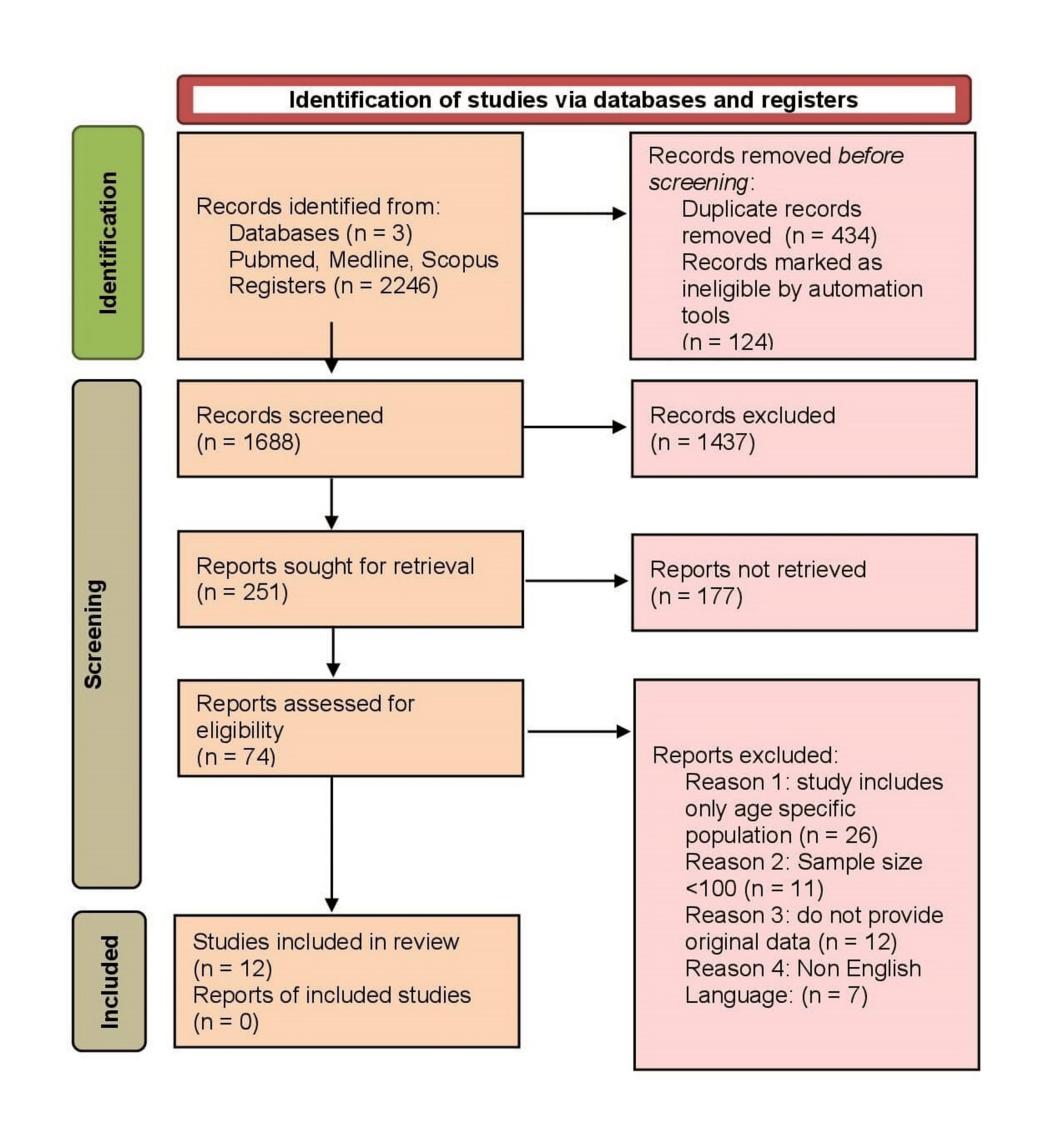
PRISMA flow chart showing identification of publications, screening, qualifying criteria, and final selection of publications for meta-analysis PRISMA: Preferred Reporting Items for Systematic reviews and Meta-Analysis

The study selection process following the PRISMA 2020 flow diagram is shown in Figure 1 in a detailed manner.

The included studies consist of prospective diagnostic accuracy studies (n=8), cross-sectional studies (n=2), case-control (n=1), and retrospective cohort (n=1).

Data Extraction and Quality Assessment

Two reviewers independently extracted data using a pre-designed and standardized extraction form. The following information was recorded from each study: a. Author(s), year of publication; b. Study design and setting; c. Population characteristics (age, menopausal status); d. Diagnostic test evaluated (CA125, HE4, ROMA, or RMI); e. Cut-off values and reference standard used; f. Diagnostic parameters: sensitivity, specificity, positive predictive value (PPV), negative predictive value (NPV), likelihood ratios (LR⁺, LR⁻), diagnostic odds ratio (DOR), and area under the ROC curve (AUC). 

If data was not available in text, graphical information was digitized using WebPlotDigitizer (v4.6). All data were extracted independently by two reviewers, and any discrepancies were resolved through discussion or adjudication by a third reviewer.

The methodological quality of the included studies was assessed using the Newcastle-Ottawa Scale (NOS) (see Table 2). The NOS was chosen because it is a validated tool suitable for assessing non-randomized and observational studies, which comprised the majority of the included literature. While QUADAS-2 is standard for randomized diagnostic accuracy studies, the NOS is more appropriate for the observational designs included in this review.

**Table 2:** Quality assessment of the studies using the NOS NOS: Newcastle-Ottawa Scale

Authors	Representativeness of the sample	Ascertainment of the malignancy	Assessment of the outcome	Statistical test	Quality Score
Oranratanaphan et al., 2018 [17]	9	9	9	9	9
Al Musalhi et al., 2016 [18]	10	10	10	9	10
Bouzari et al., 2019 [19]	10	9	10	10	9
Janas et al., 2022 [20]	10	10	9	9	9
Anton et al., 2012 [21]	10	10	9	10	10
Chawla et al., 2023 [22]	9	9	10	9	9
Arora et al., 2022 [23]	10	10	9	10	10
Winarto et al., 2014 [24]	10	10	9	9	9
Bastemur et al., 2022 [25]	9	10	9	9	9
Saleh et al., 2020 [26]	9	9	9	10	9
Laihad et al., 2013 [27]	9	9	9	10	9
Richards et al., 2015 [28]	9	9	8	8	9

Statistical Analysis

Meta-analysis was performed using R software (version 4.3.0) with the meta and mada packages. Due to heterogeneity among studies, a random-effects model (REML) was employed. Pooled estimates of sensitivity, specificity, and DOR were calculated using bivariate meta-analysis. Summary receiver operating characteristic (SROC) curves were plotted to visualize overall test performance.

Heterogeneity was assessed using the I² statistic and Cochran’s Q test. Subgroup analysis was conducted based on menopausal status. Meta-regression was not feasible due to the limited number of studies. Publication bias was evaluated using funnel plots and Egger’s test.

Ethical Consideration, Registration, and Reporting Guidelines

This meta-analysis was based solely on previously published, peer-reviewed studies available in the public domain. No human participants were involved and no individual patient data were accessed. Therefore, ethical approval was not required.

The study was not registered in PROSPERO, which is acknowledged as a limitation, as the review was initiated retrospectively.

However, to ensure methodological transparency and rigor, the study was conducted in accordance with the PRISMA 2020 (Preferred Reporting Items for Systematic Reviews and Meta-Analyses) guidelines, which were followed during the planning, screening, data extraction, analysis, and reporting phases of the review.

## Review

Results

Table 3 summarizes data from various studies included in the meta-analysis. This table emphasizes the characteristics of examining populations of post-menopausal and pre-menopausal women, with a focus on their age distribution and sample sizes. The sample sizes range from 84 to 456, thus providing a spectrum of small to moderately large datasets, crucial for ensuring statistical robustness in understanding the differences between these groups. The studies employ diverse designs such as prospective, cross-sectional, cohort, case-control, and retrospective approaches, which allow for a broad examination of population characteristics and health outcomes related to menopausal status. The mean age of participants varies significantly across studies, from 44.13 years to 75 years, reflecting the range of age groups studied, with age ranges spanning from as young as 13 years to 92 years. This diversity highlights the effort to capture a wide representation of women, both in early adulthood and advanced age, enabling a more comprehensive understanding of menopausal transition and associated factors. This shows the relevance of the sensitivity and specificity of tumor markers across different ages.

**Table 3:** Demographic features of study populations in the included studies in the meta-analysis

S. No	Author(s)	Year of Publication	Study Design	Total Sample Size	Post-menopausal	Pre-menopausal	Mean Age	Range
1	Anton et al., [21]	2012	prospective	128	73	55	45	15-90
2	Arora et al., [23]	2022	prospective analytic study	200	94	106	75	18-92
3	Bastemur et al., [25]	2022	prospective cohort study	84	44	40	54	15-74
4	Bouzari et al., [19]	2019	cross-sectional study	100	22	78	67	31 – 73
5	Chawla et al., [22]	2023	case–control study	144	66	78	67	18-82
6	Janas et al., [20]	2022	prospective	456	231	225	59	26-86
7	Laihad et al., [27]	2013	cross-sectional	128	42	86	58	13-81
8	Musalhi et al., [18]	2016	prospective, cross-sectional study	213	51	162	56	13-83
9	Oranratanaphan et al., [17]	2018	prospective analytic study	281	68	213	44.13	18–79
10	Richards et al., [28]	2015	prospective	50	29	21	56	18-68
11	Saleh et al., [26]	2020	prospective clinical study	140	30	110	64	26-69
12	Winarto et al., [24]	2014	retrospective	128	42	86	58	26-88

Diagnostic performance

The diagnostic effectiveness of CA125, HE4, ROMA, and RMI for detecting ovarian cancer was evaluated across various studies. Due to significant heterogeneity among the included studies, REMLs were employed for the meta-analysis. The diagnostic accuracy focuses on the sensitivity and specificity of CA125, with confidence intervals (2.5% and 97.5%) that estimate the precision of these measures.

Sensitivity refers to the ability of the test to correctly identify patients with the disease. In the studies, specificity refers to the ability of the test to correctly identify patients without the disease.

Studies with higher specificity are better at minimizing false positives. This further emphasizes the variation in how well the tests rule out non-diseased individuals across different research settings.

CA125

**Figure 2:**
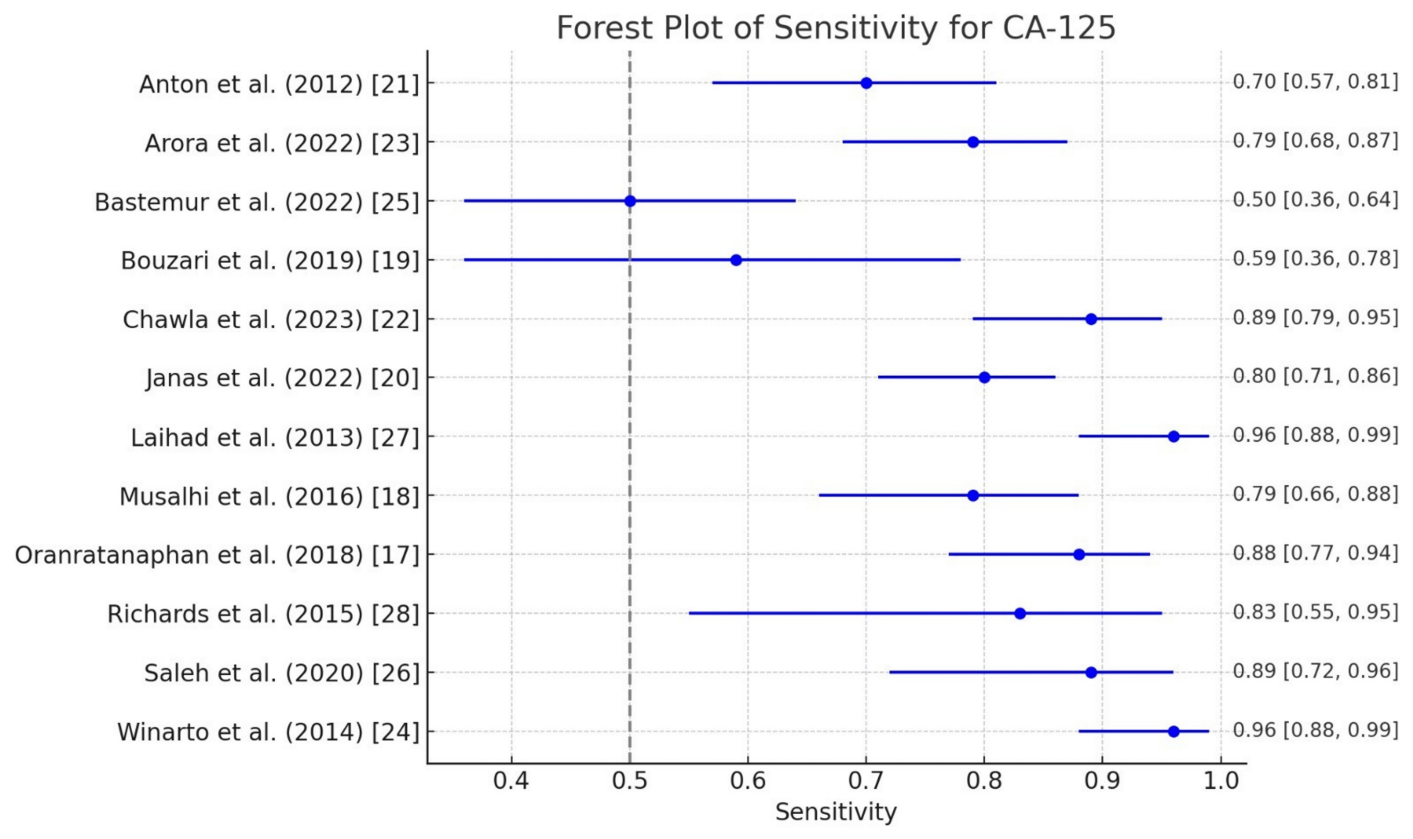
Forest plot of sensitivity for CA125 in the diagnosis of ovarian tumors CA125: Carbohydrate antigen 125

**Figure 3:**
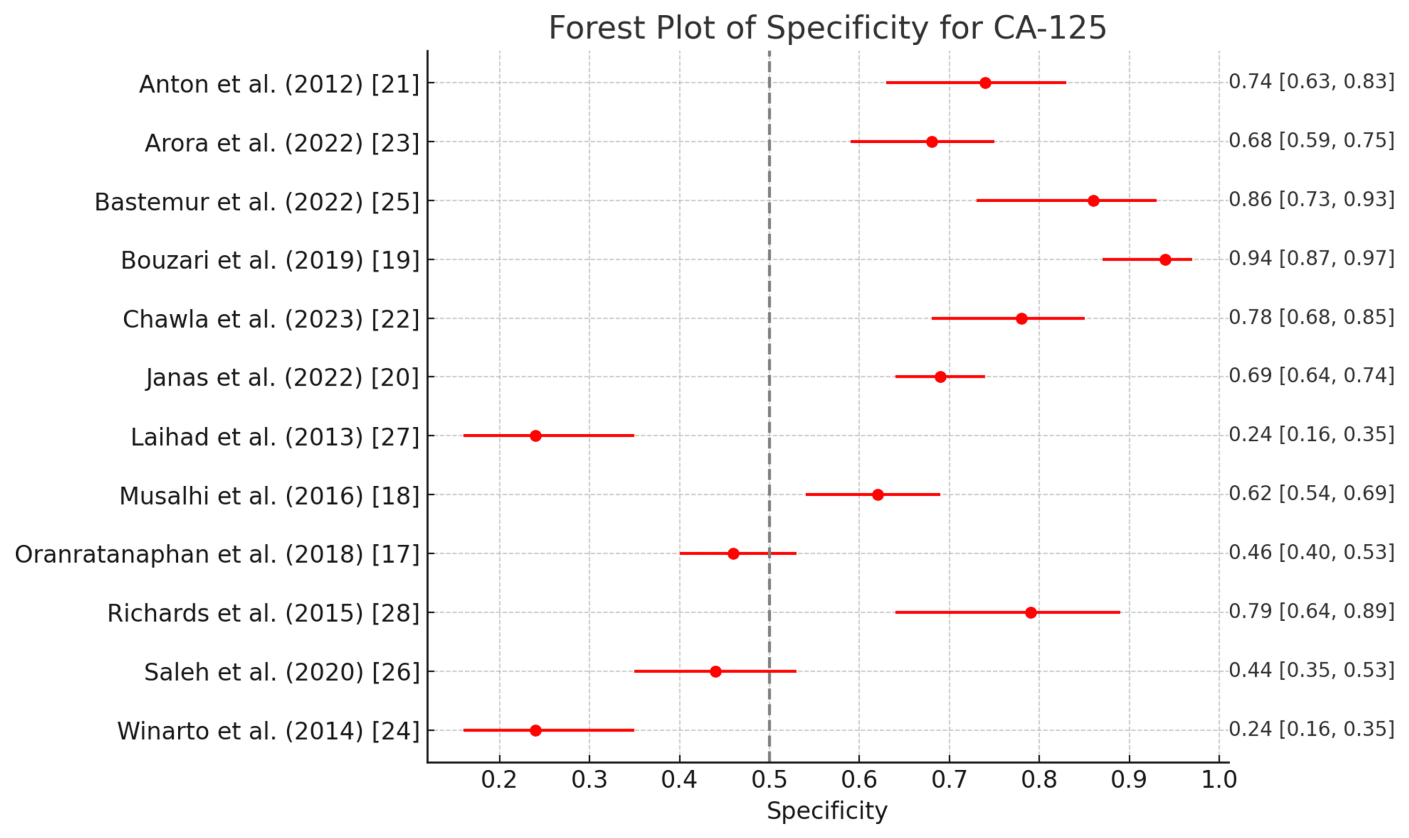
Forest plot of specificity for CA125 in the diagnosis of ovarian tumors CA125: Carbohydrate antigen 125

Figure 2 and Figure 3 outline the diagnostic accuracies of CA125, a tumor marker commonly used for ovarian cancer. Winarto et al. and Laihad et al. report the highest sensitivities 0.96 (0.88-0.99), meaning that these studies effectively detect true positives [24,27]. However, this is paired with low specificity, indicating a high false-positive rate. Studies like those by Oranratanaphan et al. and Chawla et al. also show high sensitivities of 0.88 (0.77-0.94) and 0.89 (0.79-0.95), respectively, demonstrating strong potential for CA125 in the early detection of diseases in these populations [17,22]. Bastemur et al. report the lowest sensitivity at 0.50 (0.36-0.64), indicating that the test misses many true positive cases in this study [25]. Bouzari et al. and Bastemur et al. report 0.94 (0.87-0.97) and 0.86 (0.73-0.93) specificities, respectively [19,25]. These high values suggest that the test is highly effective at correctly ruling out individuals who do not have the disease, but in some studies, this comes at the expense of sensitivity. Winarto et al. and Laihad et al. have the lowest specificities at 0.24 (0.16-0.35), meaning that these tests result in a high number of false positives, which may not be ideal in clinical practice where specificity is critical for avoiding overdiagnosis [24,27].

HE4

**Figure 4:**
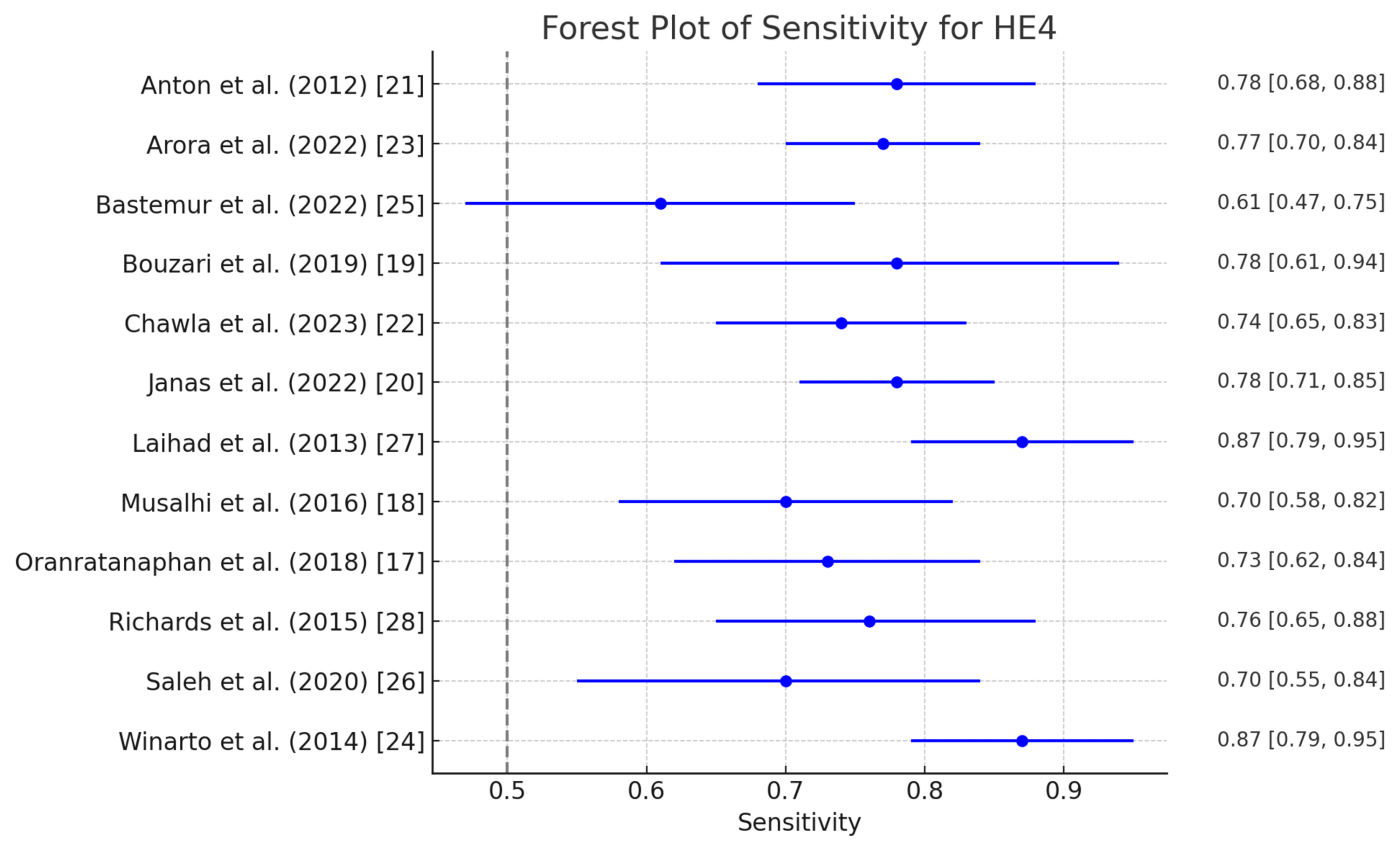
Forest plot of sensitivity for HE4 in the diagnosis of ovarian tumors HE4: Human epididymis protein 4

**Figure 5:**
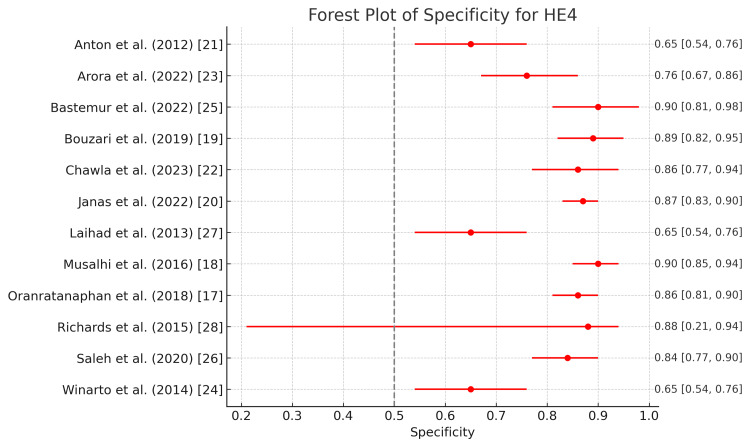
Forest plot of specificity for HE4 in the diagnosis of ovarian tumors HE4: Human epididymis protein 4

Figure 4 and Figure 5 show the different diagnostic parameters of HE4 in pre-operative diagnosis of ovarian tumors. The highest sensitivity of HE4 in diagnosing ovarian tumors was demonstrated by Laihad et al. and Winarto et al. at 0.90 (0.79-0.95), which came at a cost of specificity, as it was found to be the lowest in these two studies at 0.66 (0.54-0.76) [24,27]. This indicates the robustness of HE4 in diagnosing ovarian tumors; however, it also suggests low diagnostic accuracy of the test to detect true positives and a high number of false positives. The lowest sensitivity of HE4 was demonstrated by Bastemur et al. at 0.62 (0.47-0.75), which corresponded with the highest specificity of 0.93 (0.81-0.98), which shows the ability to correctly diagnose the true negative. Other studies also reported notable specificities of HE 4, ranging from 0.66 to 0.93 [25].

ROMA 

**Figure 6:**
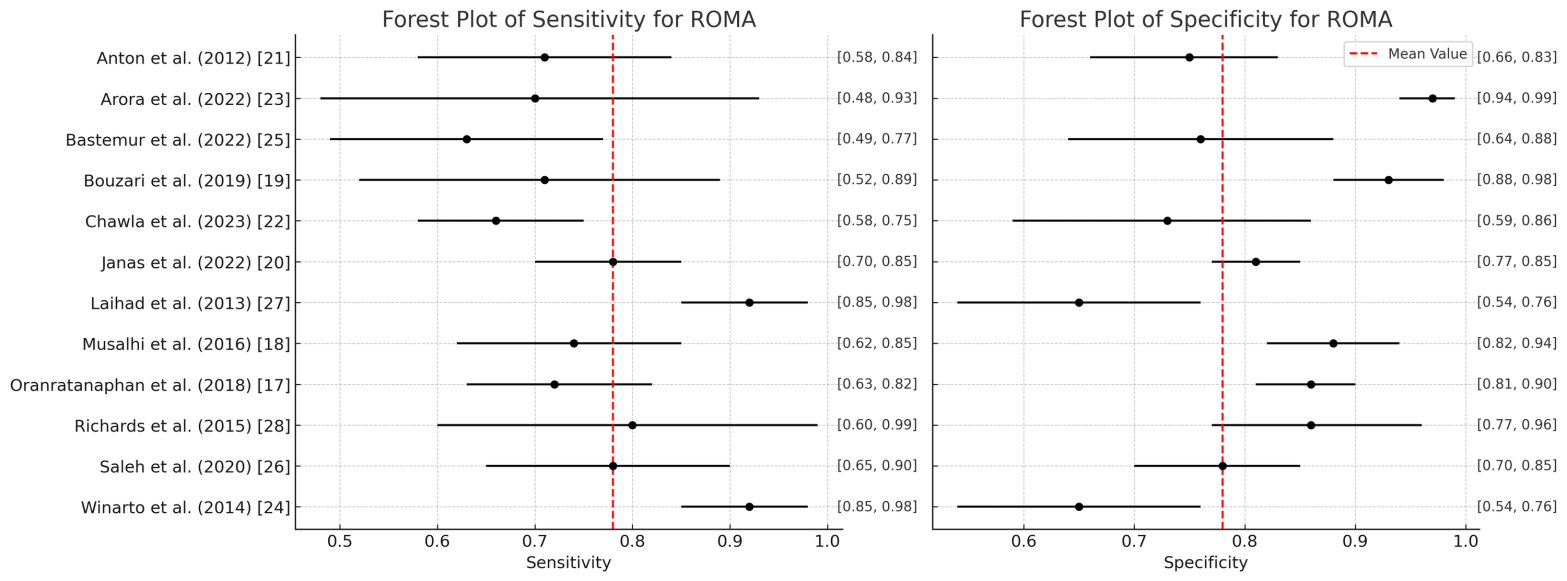
Forest plot of sensitivity and specificity for ROMA in the diagnosis of ovarian tumors ROMA: Risk of Ovarian Malignancy Algorithm

Figure 6 presents the diagnostic accuracy of ROMA based on studies assessing its sensitivity (sens) and specificity (spec) for detecting ovarian cancer. Sensitivity measures ROMA’s ability to correctly identify ovarian cancer (true positives). The sensitivity values across studies range from 0.64 to 0.94. The highest sensitivity is seen in studies by Winarto et al. and Laihad et al., both reporting a sensitivity of 0.94. Studies with lower sensitivity include Bastemur et al. (0.64) and Chawla et al. (0.671). Specificity represents ROMA’s capacity to correctly identify non-cancerous individuals (true negatives). Specificity ranges from 0.43 to 0.976 across studies. The highest specificity is seen in the study by Arora et al. (0.976) and Bouzari et al. (0.946) [19,23]. Lower specificities are reported in the study by Winarto et al. (0.43) and Laihad et al. (0.43), which suggests a higher rate of false positives in those studies [24,27].

RMI

**Figure 7:**
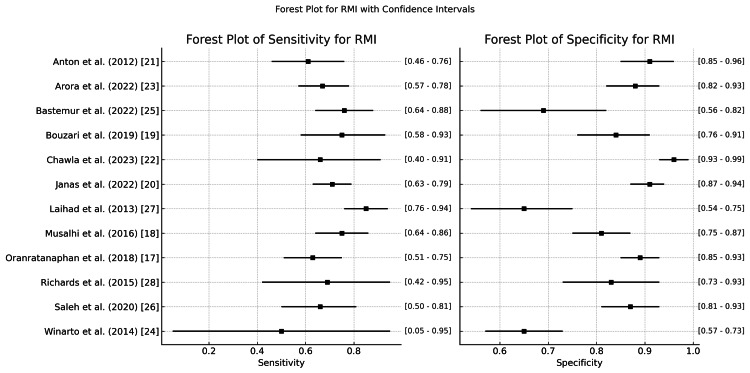
Forest plot of sensitivity and specificity for the RMI in the diagnosis of ovarian tumors RMI: Risk of Malignancy Index

Figure 7 shows a summary of the diagnostic accuracies of the RMI for various studies, focusing on their sensitivity (sens) and specificity (spec) values along with the corresponding confidence intervals. Sensitivity measures the ability of RMI to correctly identify patients with ovarian malignancy. Sensitivity ranges from 0.50 (0.05-0.95) (Winarto et al.) to 0.87 (0.76-0.94) (Laihad et al.), indicating that the RMI has variable accuracy in identifying true positives across different populations. Specificity represents RMI’s ability to correctly identify patients without ovarian malignancy. Specificity is generally high across studies, such as 0.92 (0.85-0.96) (Anton et al. [21]), and the range is typically between 0.66 (0.57-0.73) (Winarto et al. [24] and Laihad et al. [27]) and 0.97 (0.93-0.99) (Chawla et al. [22]). This indicates that the RMI tends to perform better in identifying true negatives, with some studies showing exceptional diagnostic performance.

Likelihood ratios (LRs)

LRs further refine the understanding of test performance. The positive likelihood ratio (posLR) indicates how much more likely a positive test result is to occur in individuals with the disease than those without it. On the other hand, the negative likelihood ratio (negLR) indicates the likelihood of a negative result among diseased versus non-diseased individuals. Higher posLR values (e.g., greater than 10) signify that the result significantly increases the probability of ovarian cancer. A negLR less than 0.1 suggests a strong ability to rule out the disease, making the test particularly valuable in conjunction with clinical assessments when a negative result is obtained.

CA125

**Figure 8:**
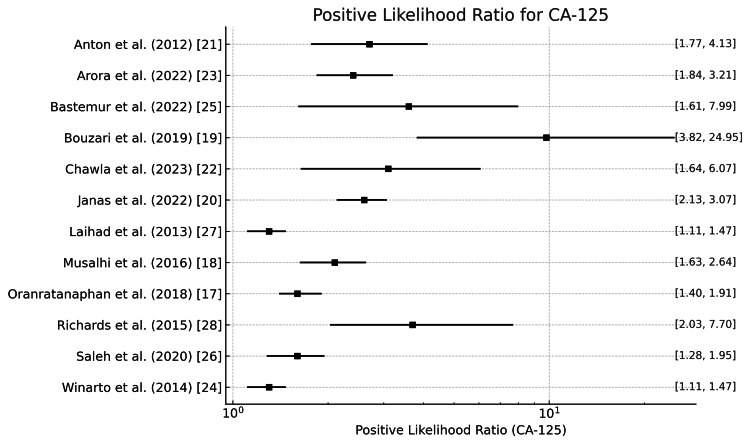
Positive likelihood ratio of CA125 in the diagnosis of ovarian tumors CA125: Carbohydrate antigen 125

**Figure 9:**
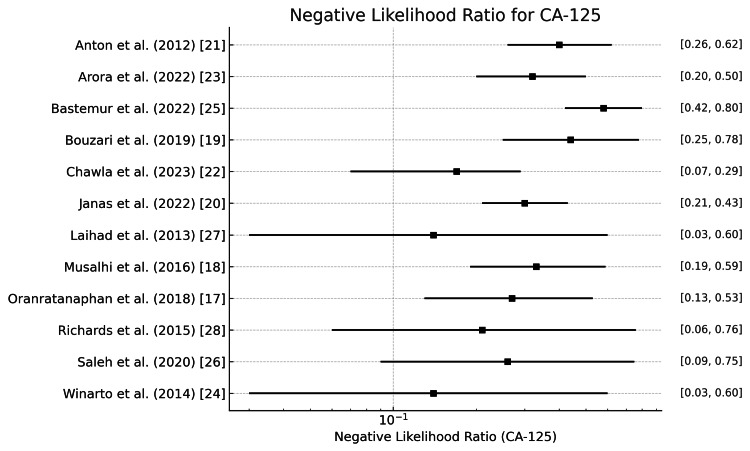
Negative likelihood ratio of CA125 in the diagnosis of ovarian tumors CA125: Carbohydrate antigen 125

The positive and negative likelihood ratios of CA 125 in diagnosing ovarian tumors are shown in Figure 8 and Figure 9. The positive likelihood ratios across studies range from 1.27 (Laihad et al. [27] & Winarto et al. [24]) to 9.76 (Bouzari et al. [19]), with a mean of 2.34. This suggests that a positive result from the CA125 test moderately increases the likelihood of ovarian cancer. The negative likelihood ratios range from 0.143 (Chawla et al. [22]) to 0.58 (Bastemur et al. [25]), with a mean of 0.284. This implies that CA125 has a good ability to rule out ovarian cancer when the test result is negative.

HE4

**Figure 10:**
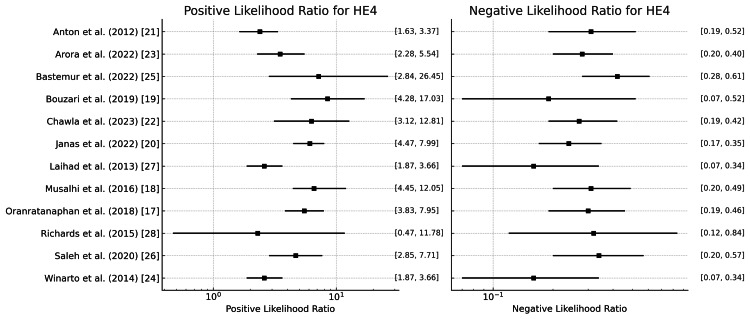
Positive and negative likelihood ratio of HE4 for the diagnosis of ovarian tumors HE4: Human epididymis protein 4

Figure 10 represents the positive likelihood ratios across studies ranging from 2.345 (Anton et al. [21]) to 8.67 (Bastemur et al. [25]), with a mean of 4.59. This suggests that a positive result from the HE4 test moderately high likelihood of ovarian cancer. The negative likelihood ratios range from 0.157 (Winarto et al. [24]) to 0.41 (Bastemur et al. [25]), with a mean of 0.271. This implies that HE4 has a good ability to rule out ovarian cancer when the test result is negative.

ROMA

**Figure 11:**
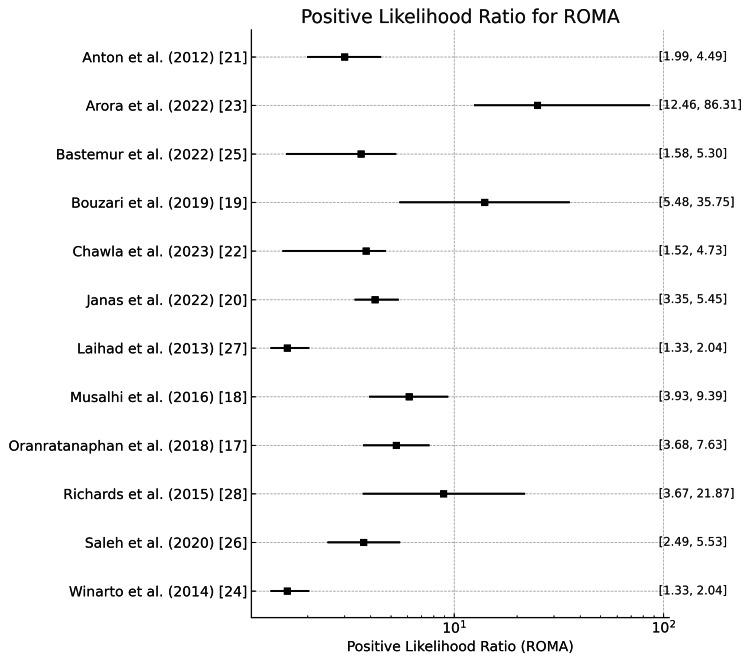
Positive likelihood ratio of the ROMA for the diagnosis of ovarian tumors ROMA: Risk of Ovarian Malignancy Algorithm

**Figure 12:**
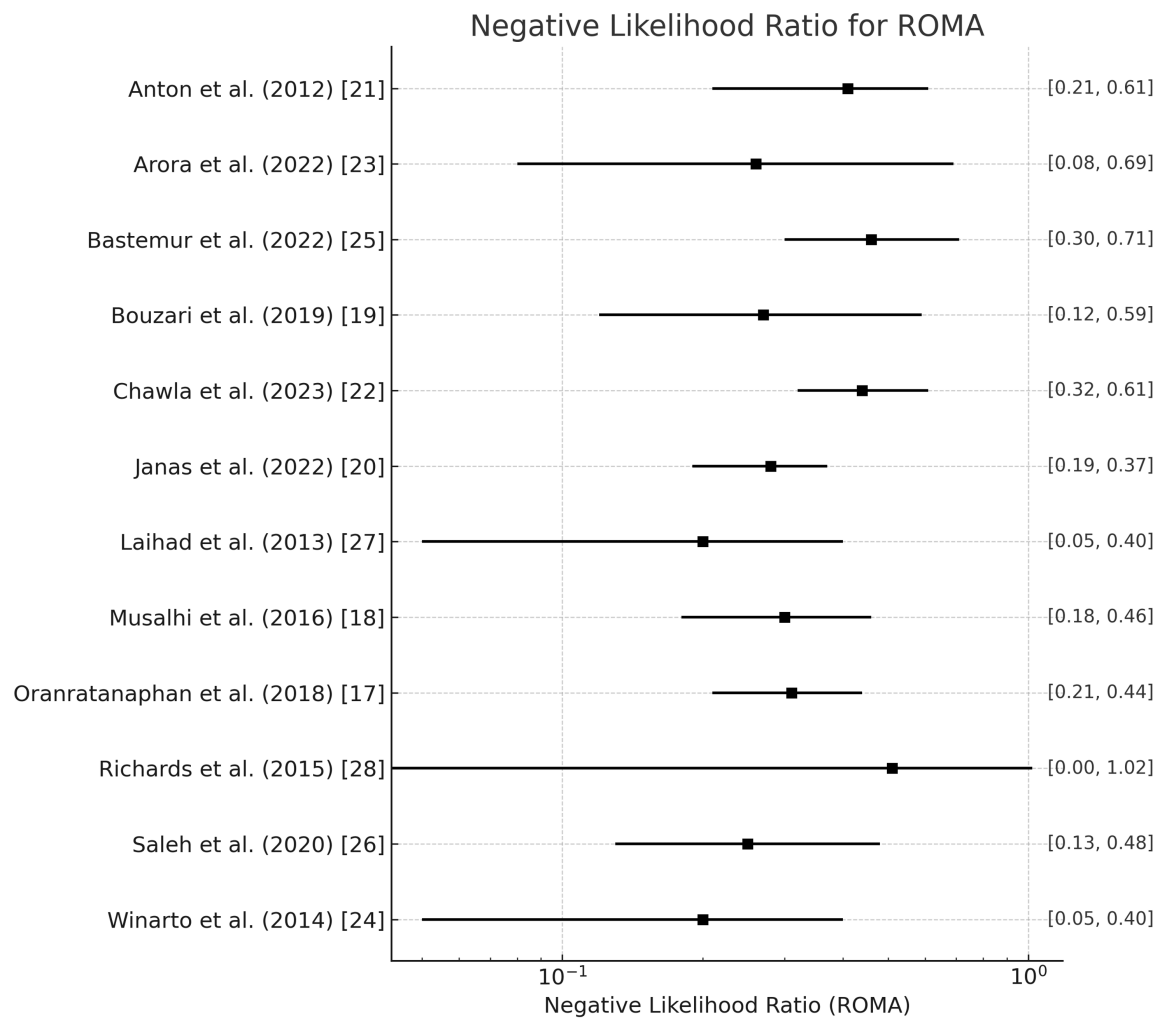
Negative likelihood ratio of the ROMA for the diagnosis of ovarian tumors ROMA: Risk of Ovarian Malignancy Algorithm

Positive and negative likelihood ratios of the ROMA in diagnosing ovarian tumors are represented in Figure 11 and Figure 12. Here, Figure 11 represents the average positive likelihood ratio across studies, which is around 4.25, with a wide range from 1.647 in the study by Winarto et al. [24] to 32.798 in the study by Arora et al. [23]. The high mean and variation in the positive likelihood ratio imply that ROMA increases the probability of ovarian cancer when positive results are obtained but is not rigid. Richards et al. reported the lowest negative likelihood ratios (0.07), making ROMA highly effective at ruling out ovarian cancer when negative [28]. In contrast, Bastemur et al. reported much higher negative likelihood ratios (0.463), with a mean value of 0.289, meaning the test is less effective in their study [25].

RMI

**Figure 13:**
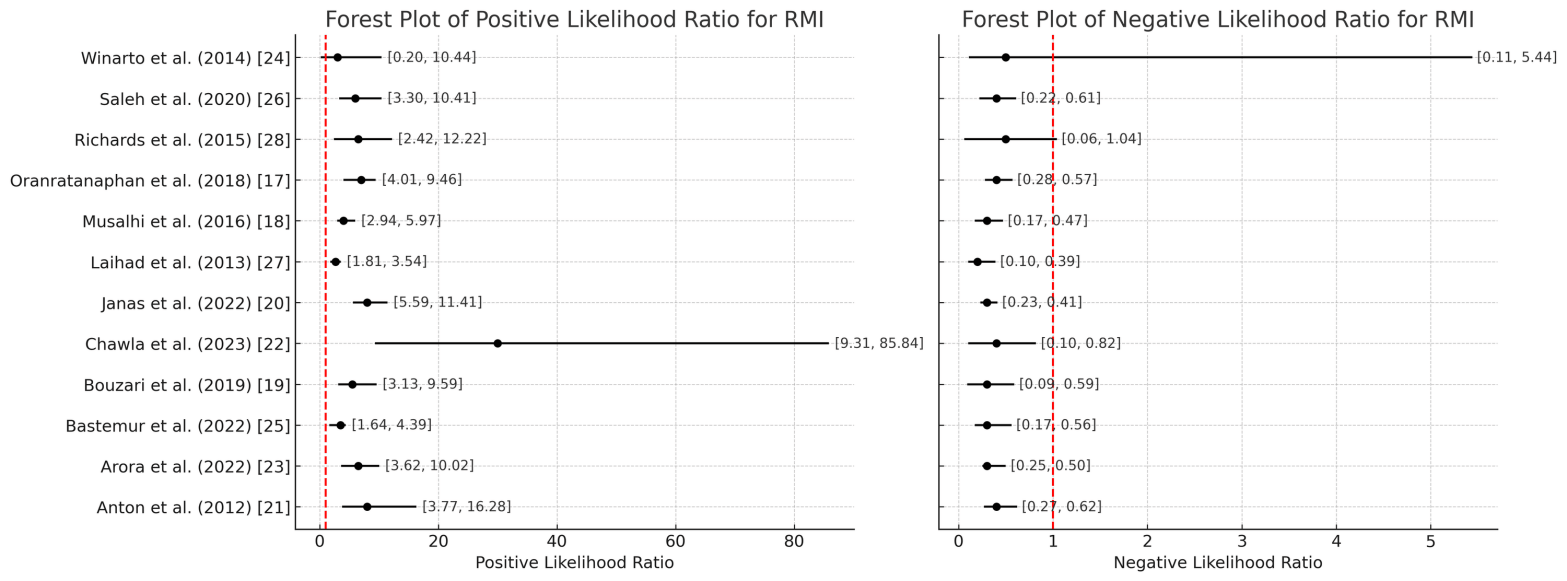
Positive and negative likelihood ratios of the RMI for the diagnosis of ovarian tumors RMI: Risk of Malignancy Index

Figure 13 represents the positive likelihood ratio values across studies ranging from 1.449 (Winarto et al. [24]) to 28.27 (Chawla et al. [22]), with a mean value of 5.150, the highest among all compared in the study; higher values suggest a stronger ability of the test to confirm the presence of the disease when the result is positive. The negative likelihood ratio values range from 0.194 (Laihad et al. [27]) to 0.763 (Winarto et al. [24]), with a mean of 0.311; this is also the highest among all higher values, increasing the risk of false positives.

Diagnostic odds ratio

The DOR combines sensitivity and specificity into a single measure, providing a more comprehensive view of a test’s diagnostic performance. A DOR greater than 1 indicates that the test is more likely to yield a positive result in patients with the disease than those without.

CA125

**Figure 14:**
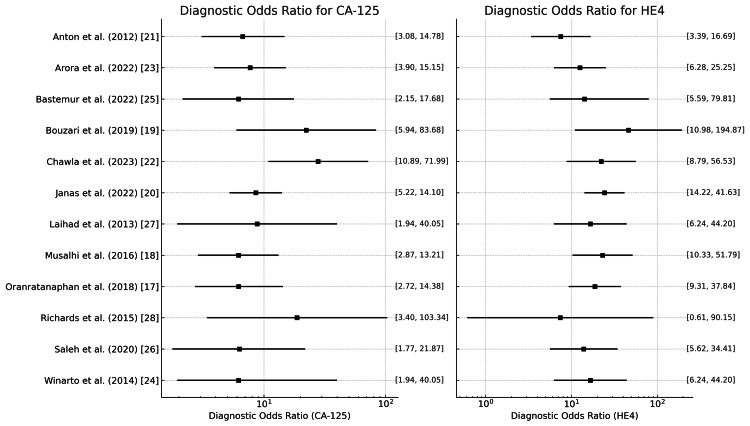
Diagnostic odds ratio for CA125 and HE4 in the diagnosis of ovarian tumors CA125: Carbohydrate antigen 125; HE4: human epididymis protein 4

The DOR, as shown in (Figure 14), ranges from 6.15 (Musalhi et al. [18]) to 28 (Chawla et al. [22]), indicating variation in the accuracy of the CA125 test across different studies. The mean DOR is 8.3, suggesting moderate diagnostic accuracy for CA125 in detecting ovarian cancer.

HE4

The DOR of HE4 is also shown in Figure 14 which ranges from 7.4 (Richards et al. [28]) to 46.25 (Bouzari et al. [19]), indicating variation in the accuracy of the HE4 test across different studies. The mean DOR is 17.0, suggesting moderate to high diagnostic accuracy for HE4 in detecting ovarian cancer.

ROMA

**Figure 15:**
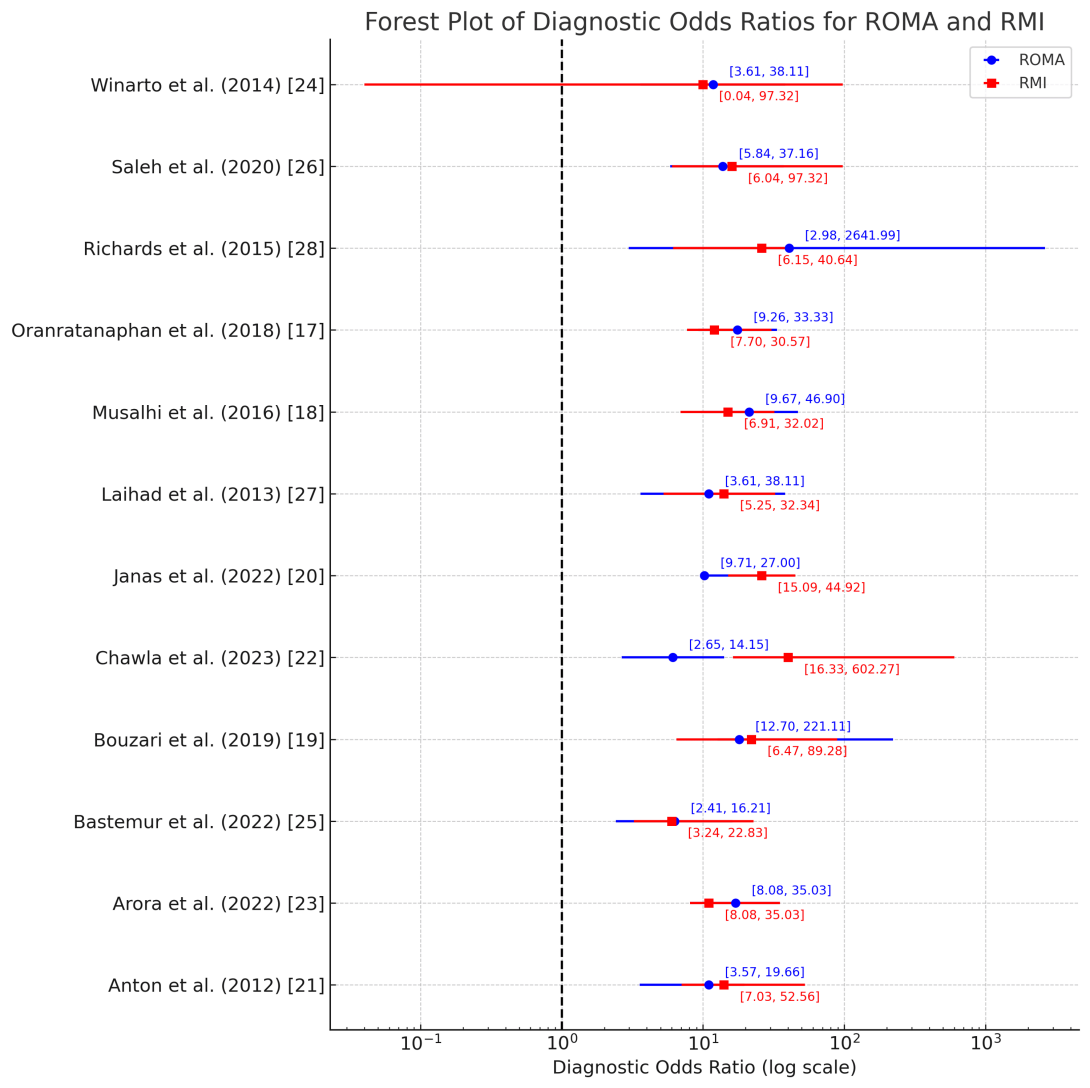
Diagnostic odds ratio for the ROMA and RMI in the diagnosis of ovarian tumors ROMA: Risk of Ovarian Malignancy Algorithm; RMI: Risk of Malignancy Index

Figure 15 shows the values of the DOR ranging from 6.13 to 140.91 with a mean of 14.8, suggesting that some studies, like that by Arora et al. [23] (DOR: 140.911), reported a much stronger diagnostic performance of the ROMA, while others, like that by Chawla et al. [22] (DOR: 6.127), had weaker diagnostic effectiveness.

RMI

Figure 15 also shows the DOR values across studies ranging significantly, from 1.9 (Winarto et al. [24]) to 99.171 (Chawla et al. [22]), indicating variability in the diagnostic performance of the test. The mean DOR across studies is approximately 16.600. The 2.5% and 97.5% confidence intervals for DOR suggest a broad range, reflecting the inherent variability in the data from different populations or methodologies.

Studies have reported DOR values ranging significantly, with some studies reflecting robust diagnostic capabilities (e.g., DORs above 20), suggesting a strong correlation between positive results and the presence of ovarian cancer. Conversely, lower DOR values might suggest less test utility in certain clinical scenarios. Based on the above result, we may conclude that HE4 performs more consistently with a mean DOR of 17.0.

SROC

The SROC curve for the diagnostic test based on a bivariate model provides a graphical representation of the overall diagnostic accuracy across multiple studies by plotting sensitivity against the false positive rate (1-specificity). The SROC curve illustrates the trade-offs between sensitivity and false positive rates across various studies. The summary estimate point on the SROC curve serves as a crucial metric. For instance, if the point lies towards the upper left of the graph (e.g., sensitivity around 0.8 and a low false positive rate), it suggests that the test is a reliable marker, effectively identifying true positives while minimizing false positives. If the SROC curve demonstrates a significant area under the curve (AUC), it indicates that the test has a good diagnostic performance. Values closer to 1 indicate excellent diagnostic accuracy, while values less than 0.5 suggest poor performance. A curve that remains relatively flat or approaches the diagonal line from the lower left to the upper right corner indicates less predictive power, suggesting that the test may be ineffective at distinguishing between those with and without the disease.

CA125

**Figure 16:**
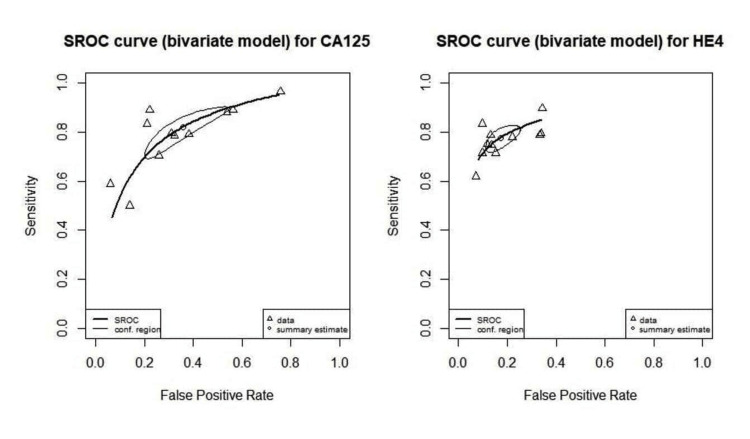
SROC curve for CA125 and HE4 in the pre-operative diagnosis of ovarian tumors CA125: Carbohydrate antigen 125; HE4: human epididymis protein 4; SROC: summary receiver operating characteristic

In Figure 16, it is shown that the SROC curve for CA125 lies toward the upper left of the graph, and the AUC value is equal to 0.811, indicating that CA125 is a reliable marker, effectively identifying true positives while minimizing false positives.

HE4

It should be noted that Figure 16 also shows that the SROC curve for HE4 lies towards the upper left of the graph, and the AUC of 0.852 signifies that HE4 is a valuable diagnostic tool for ovarian cancer, capable of effectively distinguishing between patients with and without the disease.

ROMA

The overall diagnostic performance, expressed through the AUC 0.843, indicates good diagnostic accuracy of the ROMA for ovarian cancer. Overall, the ROMA demonstrates good diagnostic ability for ovarian cancer, but its performance varies significantly across different studies. This variability suggests that the ROMA may work better in certain populations or clinical settings, highlighting the need for further validation and context-specific usage in clinical practice.

RMI

**Figure 17:**
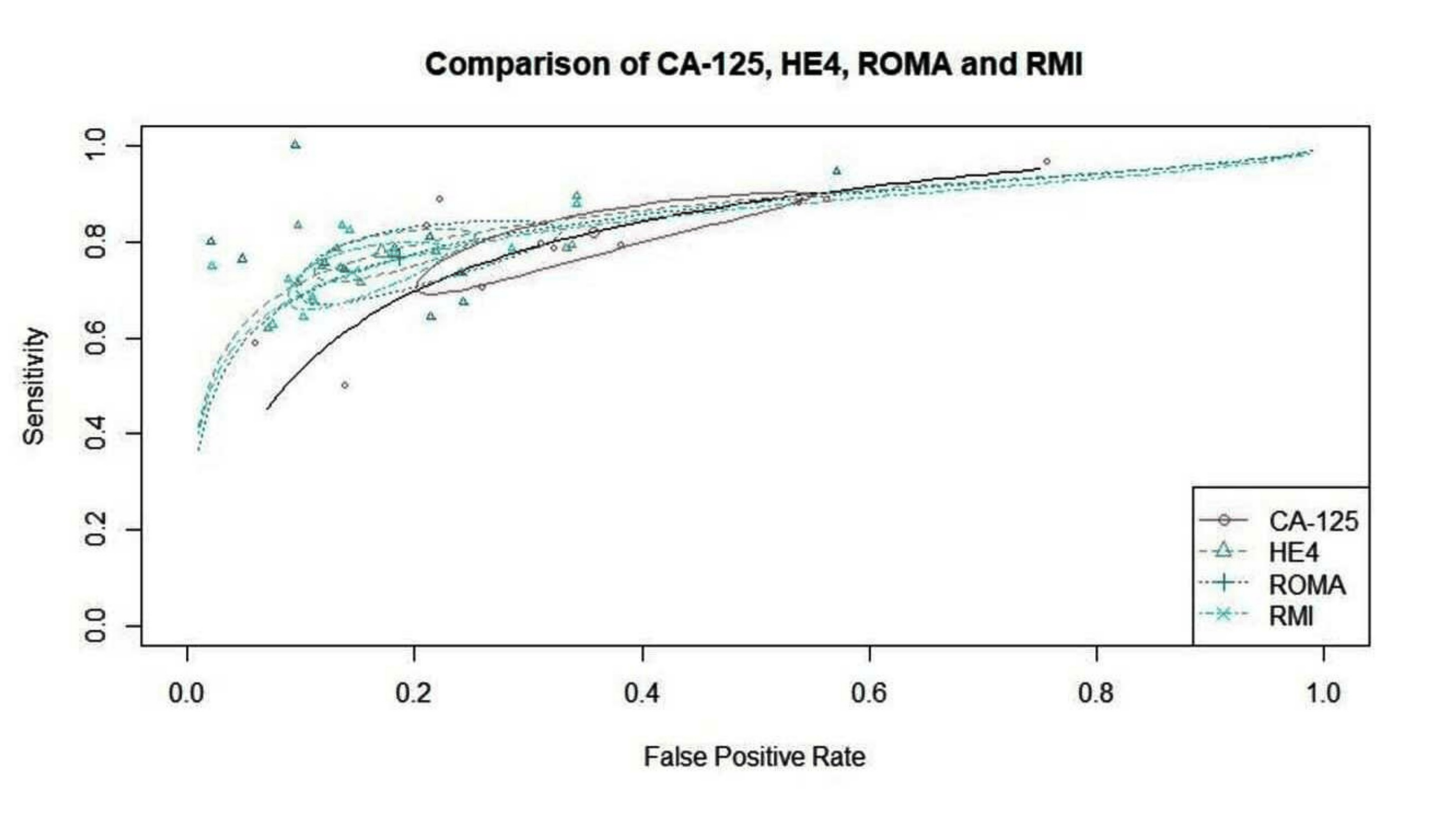
Combined SROC curve for CA125, HE4, ROMA, and RMI in pre-operative diagnosis of ovarian tumors CA125: Carbohydrate antigen 125; HE4: human epididymis protein 4; SROC: summary receiver operating characteristic; Risk of Ovarian Malignancy Algorithm; RMI: Risk of Malignancy Index

The AUC of 0.836 indicates good overall diagnostic accuracy, suggesting that the test effectively distinguishes between positive and negative cases. Figure 17 shows the combined SROC curve for CA125, HE4, ROMA and RMI in pre-operative diagnosis of ovarian tumors. The summary estimates are well separated, though the confidence regions slightly overlapped. It would however be safe to conclude that HE4 is the most reliable way to diagnose ovarian tumors with approximately the same performance as ROMA and RMI then CA125.

Discussion

Among the 12 studies included in this meta-analysis, the majority of the studies were prospective studies, but there were other studies that were cross-sectional, case-control, cross sectional, and retrospective in nature. These studies evaluated the diagnostic accuracy of CA125, HE4, ROMA, and RMI in pre-operative diagnosis of ovarian tumors. The included studies had a wide variety of population ages ranging from younger age groups starting from 13 years of age group to extreme old age of more than 90 years. The studies included have been conducted in different ethnicities around the world, thereby further strengthening the relevance of the results of this meta-analysis from populations across the globe. Using the parameters like sensitivity, specificity, true positive, true negative, a statistical analysis was done to calculate the false positive rate, odds ratio (DOR), and positive and negative likelihood ratios.

CA125

Among the studies included, the cut-off values used for CA125 were 35IU/ml in the majority of studies, but Bouzari et al. used 99.7IU/ml for CA125, whereas Bastemur et al. used 52.4IU/ml as a cut-off value [25]. The mean value of CA125 in the malignant group ranged between 298.32IU/ml (lowest in the study by Bastemur et al. [25]) to 1763.47IU/ml (highest in the study by Winarto et al. [24]). The sensitivity of CA125 in pre-operative diagnosis of Ovarian Malignancy among the pre-menopausal women was found to be 67% (lowest in the study by Saleh et al. [26] and Musalhi et al. [18]) and 93.7% (highest in the study by Chawla et al. [22]). The mean sensitivity of CA125 for all the studies included in this study was 79.90%. Bivariate analysis using the REML showed sensitivity at 0.820, false positive rate at 0.357; the estimates for the sensitivity intercept and false positive rate intercept are 1.519 and -0.587, while the AUC was found to be 0.811. To test the equality of sensitivity, a chi-square test is performed, and the calculated chi-square value is 60.22 with a p-value (8.43e-09), indicating a significant difference in sensitivity across the studies. This suggests that the performance of CA125 in detecting the condition varies significantly between studies, possibly due to differences in study design, population, or other factors. Similarly, to test the equality of specificity, a chi-square test is performed, and the calculated chi-square value is 194.31 with a p-value (<2e-16), indicating a highly significant difference in specificity across the studies. This further emphasizes the variation in how well CA125 can rule out non-diseased individuals across different research settings.

HE4

On similar lines, the cut-off values for HE4 used in the majority of studies were 70pmol/L as a broad criterion or it was segregated as 70pmol/L for premenopausal and 140pmol/L for postmenopausal women. Bastemur et al. used 83.4pmol/L as the cut-off and the value for the same was 66.95pmol/L in the study conducted by Bouzari et al. All the studies included in this meta-analysis were conducted over a population of a wide age group with a mean age ranging from 44.13 years (Oranratanaphan et al.) to 75 years (Arora et al. [23]) The mean values of HE4 in the malignant group were reported ranging from 259.90 ± 382.17 (lowest in the study by Bouzari et al. [19]) to 1338.05 (highest in the study by Winarto et al. [24]). Mean sensitivity of HE4 was reported at 74.47%, for the ROMA it is 71.46% and for the RMI it is 68.65%. The highest sensitivity for HE4 in pre-operative diagnosis of ovarian tumors was noted by Winarto et al. which was 90% across all age groups. On the other hand, the lowest sensitivity value was noted at 38% among the post-menopausal women for HE4 by Arora et al. [23]. Similarly, the bivariate analysis of HE4 showed sensitivity at 0.777, false positive rate at 0.171; the estimates for the sensitivity intercept and false positive rate intercept are 0.324 and 0.618, while the AUC was found to be 0.852. To test the equality of sensitivity, a chi-square test is performed, and the calculated chi-square value is 18.86 with a p-value of 0.0635, greater than 0.05 (LoS), indicating no significant difference in sensitivity across the studies. This suggests that the performance of HE4 in detecting the condition does not vary significantly between studies. To test the equality of specificity, a chi-square test is performed, and the calculated chi-square value is 64.85 with a p-value (1.15e-09), indicating a highly significant difference in specificity across the studies. This suggests that the HE4 test has good diagnostic power and is good at ruling out non-diseased individuals across different research settings.

ROMA

The cut-off values for the ROMA varied across all the studies, ranging from 15.14 to 28.13. The values are further divided separately for premenopausal and postmenopausal women. The lowest sensitivity for ROMA among the pre-menopausal women was noted to be 57.1%, while the highest was 93.7%, as per Chawla et al. [22] for the post-menopausal group of women, lowest sensitivity was found by Anton et al. at 72.2% [21], and the highest sensitivity was seen by Al Musalhi et al. at 93% [18].

The bivariate analysis of ROMA showed sensitivity at 0.767, false positive rate at 0.186; the estimates for the sensitivity intercept and false positive rate intercept are 0.571 and 1.027, while the AUC was found to be 0.843. Test for equality of sensitivities found that the calculated chi-square value is 31.79 with a p-value of 0.000824, indicating significant heterogeneity in the sensitivity values across the studies, meaning there is a notable variation in ROMA’s sensitivity performance. Similarly, test for equality of specificities suggested that calculated chi-square value is 194.33 with a p-value of <2e-16 also indicating significant variability in specificities across the studies. This suggests that the performance of ROMA in diagnosing ovarian cancer varies considerably across different studies, with sensitivities and specificities fluctuating based on population and methodological differences. The overall sensitivity of around 0.767 and a false positive rate of 0.186 across studies show that the ROMA test has good diagnostic power and is good to rule out non-diseased individuals across different research settings. Some studies show excellent performance (e.g., Arora et al. [23], Bouzari et al. [19]), while others report lower diagnostic accuracy (e.g., Winarto et al. [24], Bastemur et al. [25]). The significant heterogeneity in the test results underlines the need to carefully consider the context when applying ROMA in clinical practice. RMI: The RMI showed the lowest sensitivity at 63% by Anton et al. [21] among all age groups. Winarto et al. [24] and Laihad et al. [27] reported the highest sensitivity of RMI among women of all age groups, and it was found to be 88%. The analysis of RMI showed sensitivity at 0.788, false positive rate at 0.209; the estimates for the sensitivity intercept and false positive rate intercept are 0.365 and 0.725, while the AUC was found to be 0.836. Test for equality of sensitivities showed that he calculated chi-square value is 13.6268 with a p-value of 0.254, indicating that there is no significant difference in the sensitivity values across the different studies (p-value > 0.05). Similarly, test for equality of specificities found that the calculated chi-square value is 107.7606 with p-value of <2e-16 also indicating a significant difference in the specificity values across the studies (p-value < 0.05), reflecting variability in how well RMI distinguishes non-malignant cases across different populations. Overall, RMI shows moderate to high sensitivity in detecting ovarian cancer but is significantly better in specificity, meaning it is more reliable in ruling out non-cancerous cases. Variability in sensitivity and specificity across studies suggests that factors such as population characteristics or diagnostic criteria could influence RMI’s performance.

The PPV of CA125 was found to be lowest at 29.8% among all age groups, and the highest was 98% among the postmenopausal women. Similarly, the lowest PPV for HE4 among women of all age groups was found to be 59.2 (Oranratanaphan et al. [17]) and the highest was 89.6 (Bastemur et al. [25]). The NPV of ROMA ranges from 57.3% among women of all age groups (lowest by Laihad et al.) to 96% among post-menopausal women (Chawla et al. [22]). The PPV of RMI was lowest at 47.4% (Richards et al. [28]), more so in the pre-menopausal women (14.3%, Richards et al. [28]), and was highest at 80% in the study by Janas et al. [20].

When the NPV of CA125 was evaluated, it was found to be lowest at 46.2% (Oranratanaphan et al.) among women of all age groups and was highest at 94.1 % in the same age group (Saleh et al. [26]). The lowest and highest NPV of HE4 was found to be 61% (among post-menopausal women, Arora et al. [23]) and 97.52% (in women of all age groups, Janas et al. [20]), respectively.

The NPV of RMI ranges from 74.2% (all age groups, Richards et al. [28]) to 97.77% (premenopausal women, Janas et al. [20]) The AUC for CA125 was 0.938 (highest, among postmenopausal women, Al Musalhi et al. [18]) and lowest was at 0.557(postmenopausal age group, Arora et al. [23]). The highest and lowest values of AUC for HE4 were 0.909 (Janas et al. [20]) and 0.775 (Bastemur et al. [25]) among women of all age groups respectively. Similarly, the highest value for AUC of ROMA was 0.92 (among all age groups, Bouzari et al. [19]) and the lowest value was 0.749 (Bastemur et al. [25]) in the same age group.

The AUC of RMI was 0.775 (lowest, Bastemur et al. [25]) and the highest AUC was recorded at 0.895 (Janas et al. [20]) in women of all age groups.

Overall, the results of this meta-analysis suggest that CA125 has higher sensitivity in the majority of studies as compared to HE4, which was closely followed by ROMA and then the RMI. However, the specificity of HE4 was found to be higher than CA125 in most of the studies. This points towards the nonspecific nature of CA125, where it can be raised in multiple benign conditions like endometriosis, pelvic Inflammatory disease, genital TB, and others. When this was further studied to differentiate the specificity of HE4 among premenopausal and postmenopausal women, it was found that 6 out of 12 studies (Oranratanaphan et al. [17], Chawla et al. [22], Arora et al. [23], Winarto et al. [24], Bastemur et al. [25], Richards et al. [28]) showed that HE4 has higher specificity in the postmenopausal age group, and the rest showed vice versa. Ten out of 12 studies suggested that the ROMA and RMI also had higher specificity as compared to the traditionally and widely used CA125.

Limitations of the study

Despite the valuable insights provided by this meta-analysis, several limitations must be considered. One of the primary limitations is the heterogeneity in study designs, as the included studies varied in methodology (prospective, retrospective, cross-sectional, and case-control), which may introduce bias and affect the consistency of results. Additionally, there was significant variation in the cutoff values used for CA125, HE-4, ROMA, and RMI across different studies, making direct comparisons difficult and potentially influencing the reported sensitivity and specificity values. This variation also reflects real-world clinical practice. Rather than excluding these studies, we accounted for this heterogeneity through subgroup analyses and sensitivity testing wherever feasible. Importantly, this meta-analysis provides a comprehensive overview of the diagnostic performance across diverse settings and highlights the urgent need for standardized thresholds in future research. Another important limitation is the diversity in population characteristics, as the studies encompassed a wide range of age groups and ethnicities, leading to potential confounding factors such as genetic variations, environmental influences, and differences in healthcare access, which could impact diagnostic accuracy. Furthermore, most studies primarily focused on postmenopausal women, with limited data on premenopausal women, which may affect the generalizability of the findings to younger patients. The potential for publication bias is also a concern, as studies with negative or inconclusive findings are less likely to be published, potentially leading to an overestimation of the diagnostic performance of these biomarkers. However, such bias is inherent to most systematic reviews and meta-analyses. To mitigate this, we conducted a comprehensive search across multiple databases, including gray literature and conference abstracts, and performed statistical assessments for publication bias (e.g., funnel plot analysis, Egger’s test). Despite these efforts, we acknowledge that exclusion of unpublished or inaccessible negative studies remains a limitation.

Additionally, a lack of standardization in diagnostic criteria across studies, including differences in gold-standard methods for confirming ovarian cancer, may have influenced the reported accuracy rates. Lastly, although the objective of this study was to conduct a meta-analysis of existing, peer-reviewed literature, the inherent absence of unpublished studies, especially those with negative or inconclusive results, may still present a limitation. These studies, while part of the existing body of evidence, are often not publicly accessible due to publication bias. Their exclusion, not by design but by availability, could lead to an overestimation of the diagnostic accuracy of the biomarkers and may limit the overall comprehensiveness of the analysis.

Another limitation of this study is that the study was not registered in PROSPERO, as the review was initiated retrospectively. However, to ensure methodological transparency and rigor, the study was conducted in accordance with the PRISMA 2020 guidelines, which were followed during the planning, screening, data extraction, analysis, and reporting phases of the review.

## Conclusions

This meta-analysis highlights the varying diagnostic performance of CA125, HE4, ROMA, and RMI in detecting ovarian cancer. CA125 exhibited the highest sensitivity among the biomarkers, making it a valuable tool for initial screening. However, its high false-positive rate limits its specificity, indicating a risk of misclassification. In contrast, HE4 demonstrated a more favorable balance between sensitivity and specificity, reducing false-positive results while maintaining strong diagnostic performance. The RMI and ROMA also showed reliable accuracy, with ROMA emerging as the best-performing parameter in terms of overall diagnostic accuracy.

Among all parameters, HE4 exhibited the highest diagnostic odds ratio, suggesting its superior ability to differentiate between benign and malignant ovarian tumors. While CA125 remains widely used, its lower DOR highlights its nonspecific correlation with ovarian malignancy. The ROMA demonstrated the highest overall diagnostic accuracy, closely followed by HE-4 and RMI, emphasizing their potential utility in clinical decision-making. These findings suggest that a multimodal approach, integrating multiple biomarkers, may enhance diagnostic accuracy and improve patient outcomes.

## Appendices

Certainty of evidence - GRADE assessment

The certainty of evidence for each diagnostic test was evaluated using the GRADE framework. Key domains assessed were risk of bias, inconsistency, indirectness, imprecision, and publication bias. Table 4 shows the GRADE assessment of CA125, HE4, RMI, and ROMA for assessment of malignant ovarian tumors.

**Table 4:** GRADE assessment of CA-125, HE4, RMI, and ROMA CA125: Carbohydrate antigen 125; HE4: human epididymis protein 4; Risk of Ovarian Malignancy Algorithm; RMI: Risk of Malignancy Index

Diagnostic Marker	Sensitivity	Specificity	DOR	Overall Certainty
CA-125	Moderate	Low	Moderate	Moderate
HE-4	Moderate	Moderate	High	Moderate–High
ROMA	Moderate	Moderate	Moderate	Moderate
RMI	Low	Moderate	Moderate	Moderate

The GRADE evaluation supports moderate to high confidence in the findings for HE4 and ROMA, and moderate confidence in CA-125 and RMI outcomes.
